# ROR1-AS1 might promote in vivo and in vitro proliferation and invasion of cholangiocarcinoma cells

**DOI:** 10.1186/s12885-023-11412-1

**Published:** 2023-09-28

**Authors:** Xueliang Li, Zhaowei Sun, Li Wang, Qinlei Wang, Maobing Wang, Jingyun Guo, Haoran Li, MenShou Chen, Guanghua Cao, Yanan Yu, Haochen Zhong, Hao Zou, Kai Ma, Bingyuan Zhang, Guolei Wang, Yujie Feng

**Affiliations:** 1 Department of Hepatobiliary and Pancreatic Surgery, The Affiliated Hospital of Qingdao University, Qingdao City, Shandong Province 266003 China; 2 Department of Hepatobiliary and Pancreatic Surgery, HuiKang Hospital of the Affiliated Hospital of Qingdao University of Qingdao, Qingdao City, Shandong Province 266520 China; 3 Department of operating theater, the Affiliated Hospital of Qingdao University, Qingdao City, Shandong Province 266003 China; 4 Department of Internal Medicine-Cardiovascular, The Affiliated Hospital of Qingdao University, Qingdao City, Shandong Province 266003 China

**Keywords:** Cholangiocarcinoma, Immune infiltration, Enrichment analysis, Prognosis, Tumor proliferation, Migration and invasion

## Abstract

Long non-coding RNAs (lncRNAs) play important roles in many pathophysiological processes, including cancer progression. Namely, lncRNA Receptor-tyrosine-kinase-like orphan receptor-1 antisense 1 (ROR1-AS1) is crucial for cancer occurrence and progression in organs such as the liver or bladder. However, its expression and role in cholangiocarcinoma (CCA) have not been thoroughly explored.

Firstly, we assessed cell viability, proliferation, invasion, and migration using three cell lines (HuCCT-1, QBC399, and RBE) to explore the biological characteristics of ROR1-AS1 in CCA. Secondly, to determine the in vivo effect of ROR1-AS1 on tumor growth, ROR1-AS1 knockdown (KD) HuCCT-1 cells were subcutaneously injected into nude mice to evaluate tumor growth. Finally, we conducted a bioinformatic analysis to confirm the role of ROR1-AS1 in the prognosis and immunity of CCA.

In this study, we found that lncRNA ROR1-AS1 was increased in CCA samples and patients with higher ROR1-AS1 expression had a shorter overall survival period. siRNA-mediated KD of ROR1-AS1 significantly reduced cell proliferation and inhibited the migration of CCA cells. In addition, ROR1-AS1 KD HuCCT-1 cells injected into nude mice grew slower than normal CCA cells.

In summary, our results show that ROR1-AS1 can promote CCA progression and might serve as a new target for diagnosis and treatment of CCA.

**Supplementary Information:**

The online version contains supplementary material available at 10.1186/s12885-023-11412-1.

## Introduction

Cholangiocarcinoma (CCA) is a group of malignancies that can arise anywhere in the biliary system [1]. This aggressive malignant tumor has poor patient outcomes even when diagnosed at an early stage [2]. Biliary tract malignancies, particularly CCAs, are rare tumors with serious consequences [3]. Furthermore, changes in cancer-related genes and inflammation-related signaling pathways have been implicated in CCA development [4].

Receptor-tyrosine-kinase-like orphan receptor-1 (ROR1) promotes further development of neurites, as it supports the survival of receptor tyrosine kinase-like orphan receptors. It is a glycosylated type I membrane protein that is part of the receptor-tyrosine-kinase-like orphan receptor (ROR) subfamily of cell surface receptors [5, 6]. ROR1 antisense RNA 1 (ROR1-AS1) is a newly discovered long noncoding RNA (lncRNA) located at 1p31.3 of the human genome. This lncRNA participates in the regulation of gene transcription in combination with the polycomb inhibitory complex 2, which was first reported in 2017, and may be a neoplasm biomarker for patients with mantle cell lymphoma [7].The lncRNA ROR1-AS1 has the ability to regulate certain tumors as one of the particular sections of ROR1, which could modulate some human diseases and biological behaviors. For example, ROR1-AS1 can affect disease progression [7]. Clinical research has indicated that ROR1AS1 overexpression is associated with distant tumor metastasis and lower five-year survival rates [8]. Recently, some studies identified ROR1-AS1 as a biomarker, which can indicate translation results in liver cancer [9]. In lung adenocarcinoma samples, ROR1-AS1 expression levels are notably higher than those in surrounding tissues, indicating its close relation to disease progression [10]. Nevertheless, whether it can serve as a particular marker of CCA progression requires further investigation.

## Materials and methods

### Data collection

We capture relevant RNA sequencing data and corresponding patient clinical information in the TCGA data repository (https://portal.gdc.cancer.gov/repository). It involves 36 CHOL samples and 9 normal samples. The workflow type is HTSeq-TPM. We then analyze these data in the R language.

### Correlation analysis of ROR1-AS1 expression and immune-infiltrating cells in CCA

Many studies have explored the relationship between certain genes and immune-infiltrating cells to identify potential therapeutic targets for CCA. The mRNA levels of ROR1-AS1 in fresh cholangiocarcinoma tissues were examined using RT quantitative PCR (RT-qPCR). In this study, RT-qPCR was performed by using a PCR master mix and a 7300 Real-Time PCR System to estimate the expression levels of ROR1-AS1. TIMER is an informatic tool that can conduct integration analyses for tumor-infiltrating immune cells [11]. We investigated the behavior of ROR1-AS1 in diverse immune-infiltrating cells using TIMER.

### Gene set enrichment analysis

The correlations between ROR1-AS1 expression and CCA was explored using R. We conducted Gene Set Enrichment Analysis (GSEA) and Gene Ontology (GO) analyses using h.all.v7.0. symbols.gmt [hallmarks] as a reference gene dataset. Finally, R analysis was used to identify pathways that could be enriched in CCA. Genes with |ES|>1, P < 0.05, and false rate discovery < 0.25 were considered differentially expressed. To explore the mechanisms behind the progression of cholangiocarcinoma in ROR1-AS1, we conducted western blot experiments to validate our research.

### Cell culture

Three CCA cell lines were purchased: HuCCT-1, RBE, and QBC939. Cells were kept in a 37 °C, 5% CO_2_ incubator using DMEM (Gibco, USA) medium with 10% fetal bovine serum (FBS), and 1% penicillin-streptomycin.

### Cell transfection and quantitative polymerase chain reaction

QBC939, HuCCT-1, and RBE cells were seeded in Petri dishes and grown to 50–70% confluency. Cells were transfected with the following small interfering RNA (siRNAs): synthetic si-NC, si-ROR1-AS1, si-ROR1-AS2, and si-ROR1-AS3. Medium was replenished 6 h after transfection. Forty-eight hours after transfection, transfected cells were collected and RNA was collected for quantitative polymerase chain reaction (qPCR) experiments, and transfection efficiency was determined according to qPCR results.

### Cell viability and proliferation assays

The Enhanced Cell Viability Assay Kit (CCK-8) was used to evaluate the effects of ROR1-AS1 on cell growth and proliferation. Using the three transfected cell lines and normal cells as a control, cell suspensions of 2000 cells/100 µL per well in a 96-well plate were cultured at 37 °C in a humidified chamber with 5% CO_2_. After the cells adhered, CCK-8 was added to each well at 0 h, 24 h, 48 h, 72 h, and 96 h and incubated for 2 h. Absorbance was measured at 450 nm using an enzyme-labeling instrument.

For quick and sensitive EdU labelling to monitor cell proliferation, Alexa Fluor 555 was used. First, we labeled EdU and cultured the three transfected cell lines and the corresponding normal CCA cells in 6-well plates. Subsequently, we prepared an educational work solution to complete educational labels. After fixation and washing, EdU was detected. Next, we kept the cells for 30 min at room temperature in the dark, removed the click reaction solution, washed with detergent, and stained the nuclei after. Finally, cell suspensions obtained labelled by EdU or nuclear staining were assessed by flow cytometry to detect the effects of the transfected siRNAs on cell proliferation.

### Transwell and migration experiments

Transwell assays were performed to explore the role of ROR1-AS1 in cell migration and invasion. These experiments differ in the use of the Transwell and Matrigel, with the Transwell being only used for 1 h with no Matrigel in the migration experiment. For the Transwell migration experiments, we first established a migration model and then added complete medium containing 10% FBS to the lower chamber of the wells of a 24-well plate, followed by the addition of the cell suspension within the chamber. The chambers were transferred to 24-well plates containing intact medium and cultured in an incubator for at least 24 h. After incubation, the plates were removed, placed in 10% formaldehyde solution for 30 min at room temperature, washed with PBS, and stained for 1 h at room temperature. Finally, at least five random fields were analyzed and counted under a microscope.

For the Transwell invasion assay, we pre-coated the chamber with Matrigel. After 2 h at 37 °C, the cells in the prepared serum-free medium were added to the upper chamber and a medium containing 10% FBS was added to the bottom chamber as an attractive substance. The preparations were then cultured at 37 °C and 5% CO_2_. After 24 h of incubation, the cells infiltrated into the lower chamber. Cells were fixed with 10% formaldehyde under the membrane for 30 min in a normal environment and stained for 1 h. Finally, at least five random fields were counted under a microscope.

In the scratch test, cells were seeded in 6-well plates and, when the cells reached 80% confluency, the medium was switched to a serum-free medium and cells were starved for 24 h. Then, we used a 200 µL plastic pipette tip to draw a straight line with a ruler. Cells were then washed with PBS and the initial scratches were imaged. The serum-free medium was then added to the cells and they were placed in an incubator. Cells were observed under a microscope every 12–24 h and imaged for further analysis. Cell migration rate was calculated as follows: cell migration rate = (initial scratch width-final scratch width)/initial scratch width × 100%.

Finally, we used relevant software to analyze the changes in cell coverage, in order to compare the migration rates of the different cell types.

### Animal experiments

The Animal Experimental Ethics Committee of the Affiliated Hospital of Qingdao University approved the animal studies and we did our best to alleviate animal suffering. Nude mice (4 weeks old) were provided by Shandong Teke Biotechnology Company (Shandong, China). All mice were raised in a pathogen-free animal facility and randomly assigned to either the control or experimental groups (three mice per group). Using the abovementioned cell lines, mice in the experimental group were injected ROR1-AS1 knockdown (KD) cells that were knocked out for ROR1-AS1, while mice in the control group received ROR-AS1 normal cells. CCA HuccT-1 cells (3 × 10^6^) were injected in a 0.2 mL DMEM suspension into the right side of each nude mouse. After completion of the injection, we measured tumor changes every 3 days and calculated the tumor volume as V = 0.5×L×W ^2^(V = volume, L = length, W = width). After 35 days, the mice were euthanized under anesthesia, and the tumors were removed. Tumor sizes were compared to determine the effect of ROR1-AS1 in tumor progression. The list of animal studies can be seen in Supplemental Data S1. Before sacrifice, all mice were anesthetized with gaseous isoflurane at a concentration of 3.0% for induction and 1.0% for maintenance. Subsequently, the mice were sacrificed by rapid cervical dislocation. Mice with relaxed muscles without breathing or nerve reflexes were considered dead. The maximum tumor diameter was set to 2 cm. Finally, tumor tissues were prepared for histological examination by immunohistochemistry (IHC) of the Ki-67 protein.

## Results

### Correlation analysis of ROR1-AS1 expression levels in CCA

**Fig. 1:**
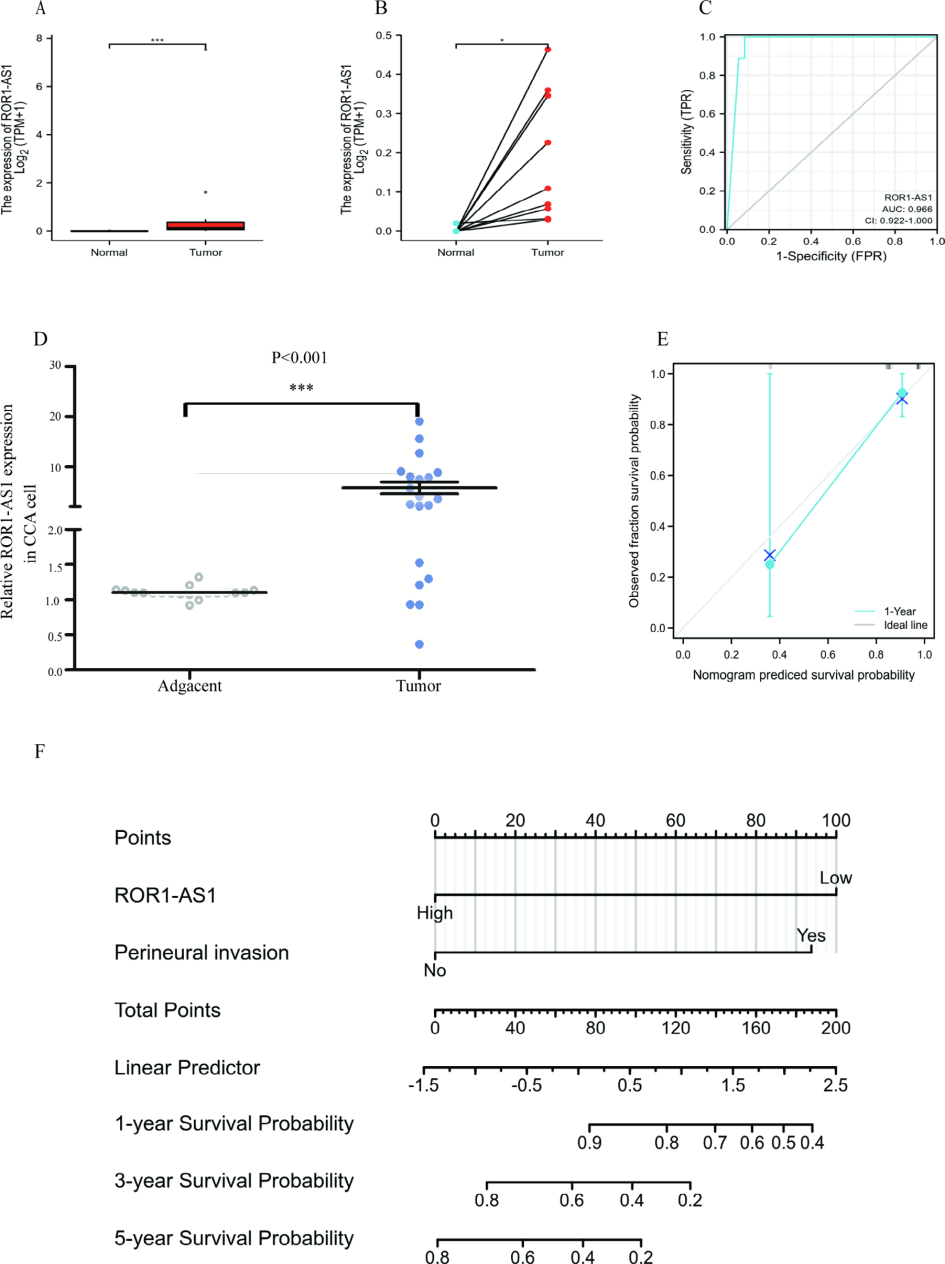
The effect of ROR1-AS1 on CCA cells proliferation and correlation analysis of ROR1-AS1 expression levels in CCA. (**A**) Relative expression levels of ROR1-AS1 in paired CCA samples. (**B**) Relative expression levels of ROR1-AS1 in un-paired CCA samples. (**C**) For estimating tumor and normal outcomes, the predictive value of ROR1-AS1 has a high accuracy (AUC = 0.966, CI = 0.922–1.000)( Results are shown as the mean ± SD. *p < 0.05, **p < 0.01, ***p < 0.001.). (**D**) The ROR1-AS1 expression level of in 20 fresh adjacent non-tumor tissues and 20 bile duct carcinoma by RT-qPCR test. (**E**) The calibration curve of the nomogram. CCA(cholangiocarcinoma) TCGA(the cancer gene atlas). (**F**) Nomogram integrating the expression of ROR1-AS1 and other factors for prognosis assessment in CCA from TCGA data

Studies have shown that ROR1-AS1 is highly expressed in numerous tumors. Although some studies have shown that the lncRNA ROR1-AS1 is overexpressed and has a prognostic value in hepatic carcinoma and colon adenocarcinoma [12, 13], there has been no research on the expression of ROR1-AS1 in CCA. As shown in Fig. 1A and B, our univariate correlation analysis revealed that ROR1-AS1 expression was significantly higher in CCA paired (P = 0.011) and unpaired specimens (P < 0.001). The expression of ANCR was measured by qRT-PCR in 20 CHOL tissues and paired normal tissues. Consistent with the data analysis in TCGA, our qPCR experiment found significantly lower expression in CHOL tissues than in the matching non-cancerous tissues (P < 0.001, Figure D).The predictive value of ROR1-AS1 expression had a high accuracy for estimating tumor and normal outcomes, as shown by an area under the receiver operating characteristic (ROC) of 0.922–1.000 (Fig. 1C). Therefore, we conclude that ROR1-AS1 may be an important diagnostic marker for CCA. We then created a nomogram of overall survival to jointly assess *CCA* and other indicators, including pathological stage, sex, and expression level (Fig. 1F), with a higher score on the nomogram indicating a worse prognosis. The calibration curve displays the nomogram’s performance for ROR1-AS1; we used the nomogram to carry out 200 bootstrap resamples and recalculated 16 samples per group, as shown in Fig. 1E.

### In vitro ROR1-AS1 overexpression and inhibition in CCA

**Fig. 2:**
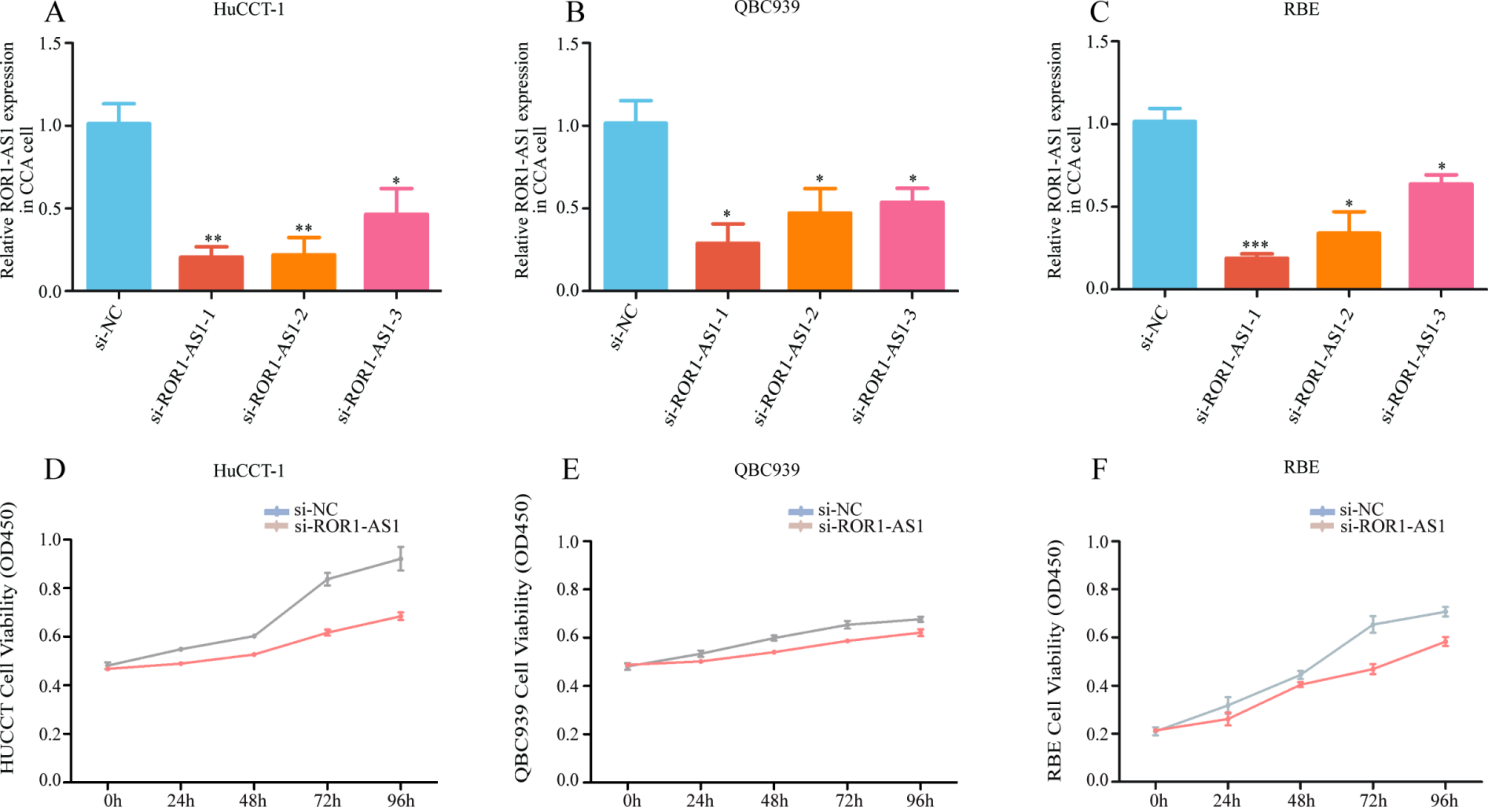
(**A-C**) The transfection efficiency of si-RNA for ROR1-AS1 inhibition in HuCCT-1, QBC939, RBE cell lines and normal CCA cells was confirmed by reverse transcription quantitative PCR analysis. (**D-F**) Compared with the corresponding control, the three ROR1-AS1 knockdown cell lines showed inhibited cell proliferation compared with normal CCA cells

The possible clinical significance of ROR1-AS1 prompted us to investigate its mechanism in CCA. Therefore, we performed experiments to verify the association between ROR1-AS1 and CCA cells. To study the location of ROR1-AS1 in CCA, si-ROR1-AS1, si-ROR1-AS2, and si-ROR1-AS3 were transfected into QBC939, HuCCT-1, and RBE cells to KD ROR1-AS1 expression. The transfection effect was confirmed by qPCR and the final experimental data showed an appropriate ROR1-AS1 KD (Fig. 2A, B, C).

Next, we transfected ROR1-AS1 KD RBE, HuCCT-1, and QBC939 cells and found that these three cell lines showed decreased proliferation (Fig. 2D, E, F).

### ROR1-AS1 knockdown inhibits CCA cell proliferation, invasion, and migration

**Fig. 3:**
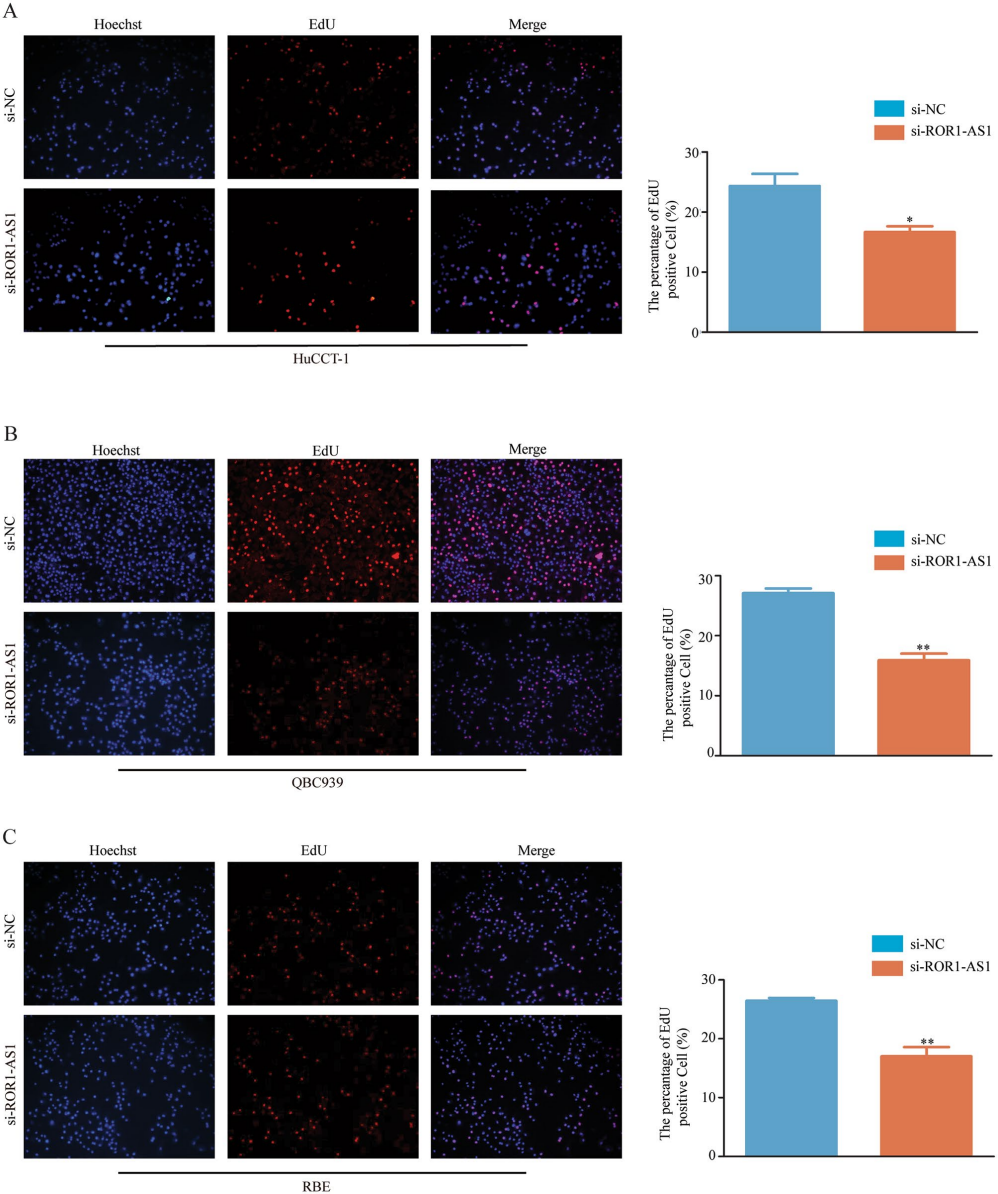
Edu experiment. (**A-C**) The three ROR1-AS1 knockdown cell lines and normal CCA cells were stained with Edu. Staining results are displayed in as graphics, while the comparison of the number of stained cells is displayed as an histogram

The transfected cells showed a relative decrease in expression compared to non-transfected cells. The CCK-8 assay showed that overexpression of ROR1-AS1 increased the proliferation of CCA cells, whereas ROR1-AS1 KD inhibited the proliferation of CCA cells at 48 h, 72 h, and 96 h. Consistently, the EdU proliferation test also showed that the proliferation of QBC939, RBE, and HuCCT-1 cell lines transfected with ROR1-AS1 was significantly reduced, in contrast to the non-transfected cells (Fig. 3A, B, C).

**Fig. 4:**
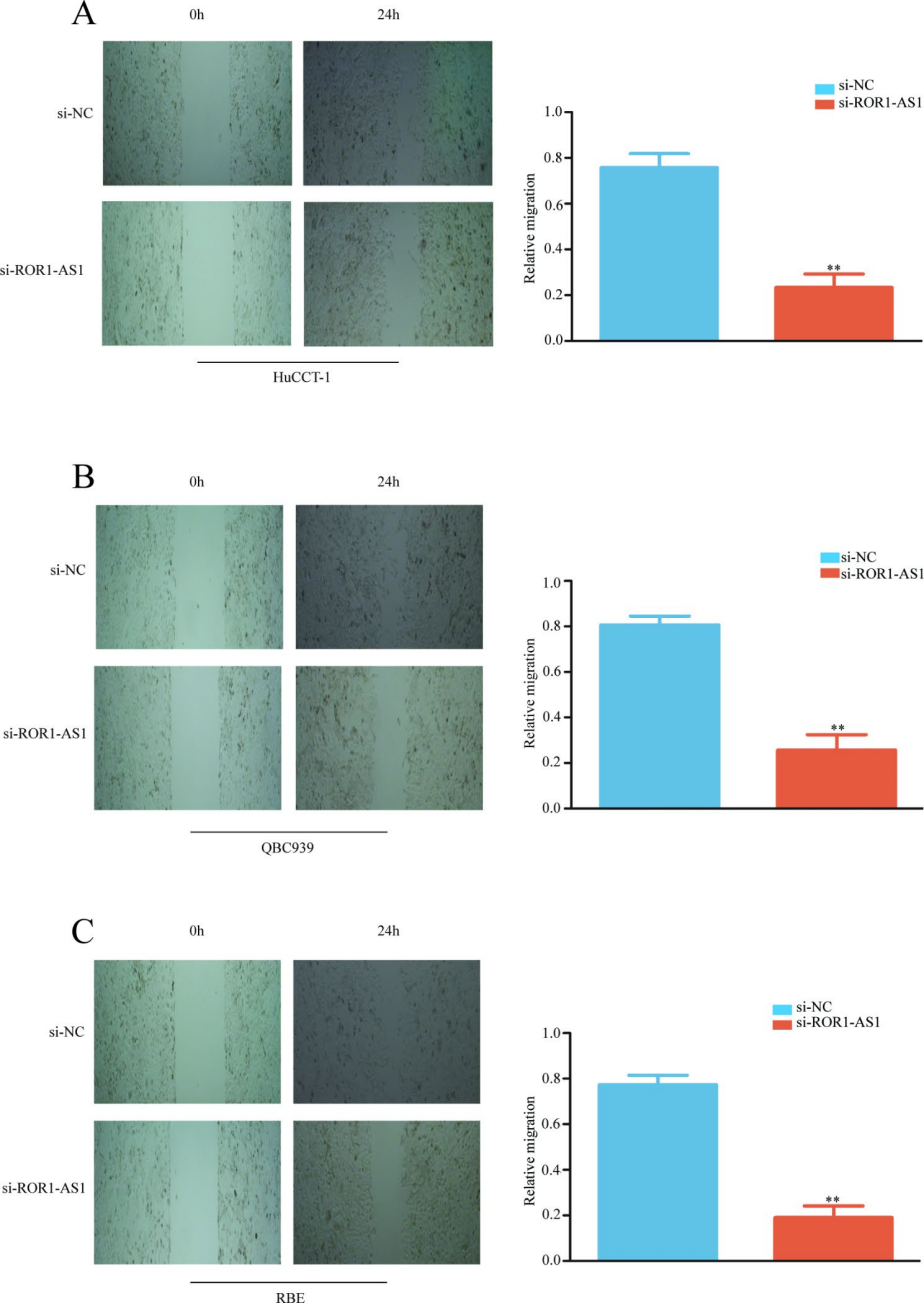
Scratch, migration, and invasion of RBE, HuCCT-1, and QBC939 cells. (**A-C**) Scratch test showed that the three ROR1-AS1 knockdown cell lines migrated slower than normal CCA cells. Histogram clearly shows the ratio of the three cell lines to normal CCA cells

**Fig. 5:**
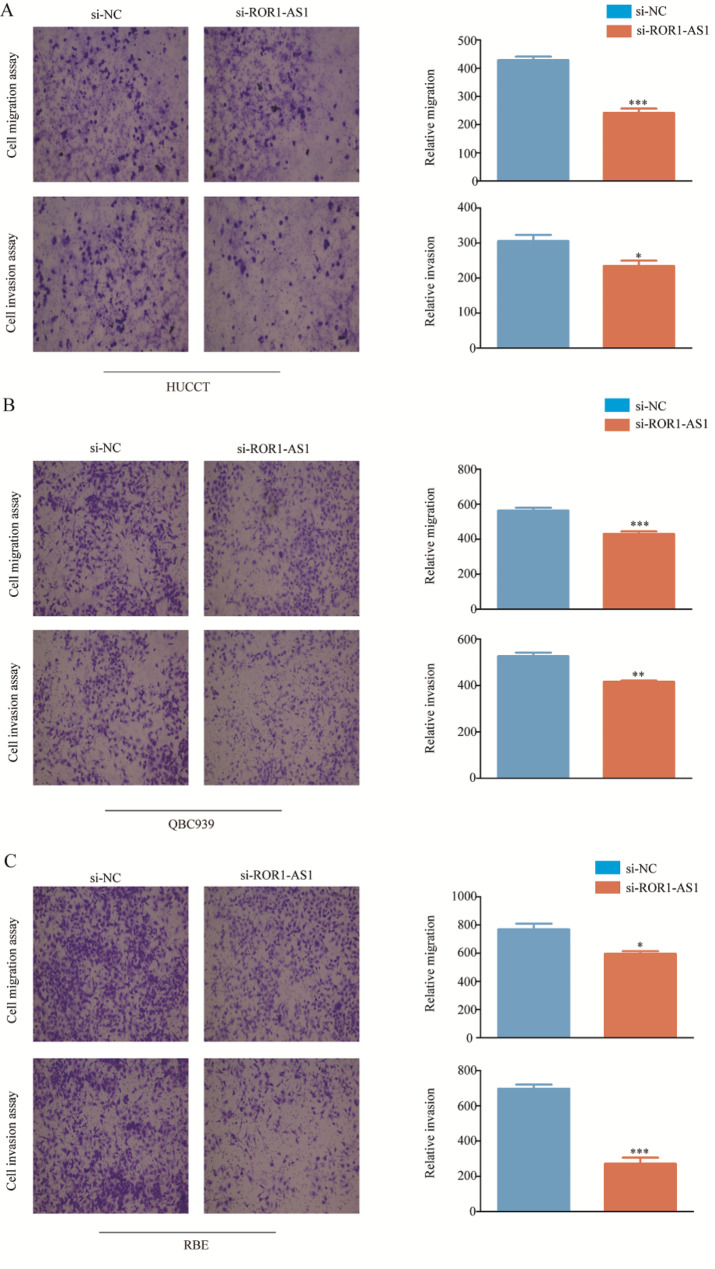
(**A-C**) Above, representative images of the number migrating of ROR1-AS1 knockdown cells and normal CCA cells migrating. Below, representative image of the corresponding number of invading cells. Quantitative measurements of migratory and invasive cells. Transwell invasion and migration tests were carried out with the three kinds of cells in the figure and the results quantified

Then we investigated the ability of ROR1-AS1 to target CCA cell migration and invasion, exploring the function of ROR1-AS1 on CCA cell migration and invasion, and comparing normal CCA cells with ROR1-AS1 KD cells. The experimental outcomes indicated that the ROR1-AS1 KD CCA cells migrated less than the non-transfected CCA cells. Consistently, the scratch assays also clearly showed that the migration ability of the transfected cells was lower than that of non-transfected CCA cells (Fig. 4A, B, C), and the number of invading cells in the si-ROR1-AS1 group was significantly lower than that of the si-NC group(Fig. 5A, B, C). Therefore, we concluded that ROR1-AS1 plays a facilitation role in migration and invasion of CCA cells.

### In vivo ROR1-AS1 role in CCA

**Fig. 6:**
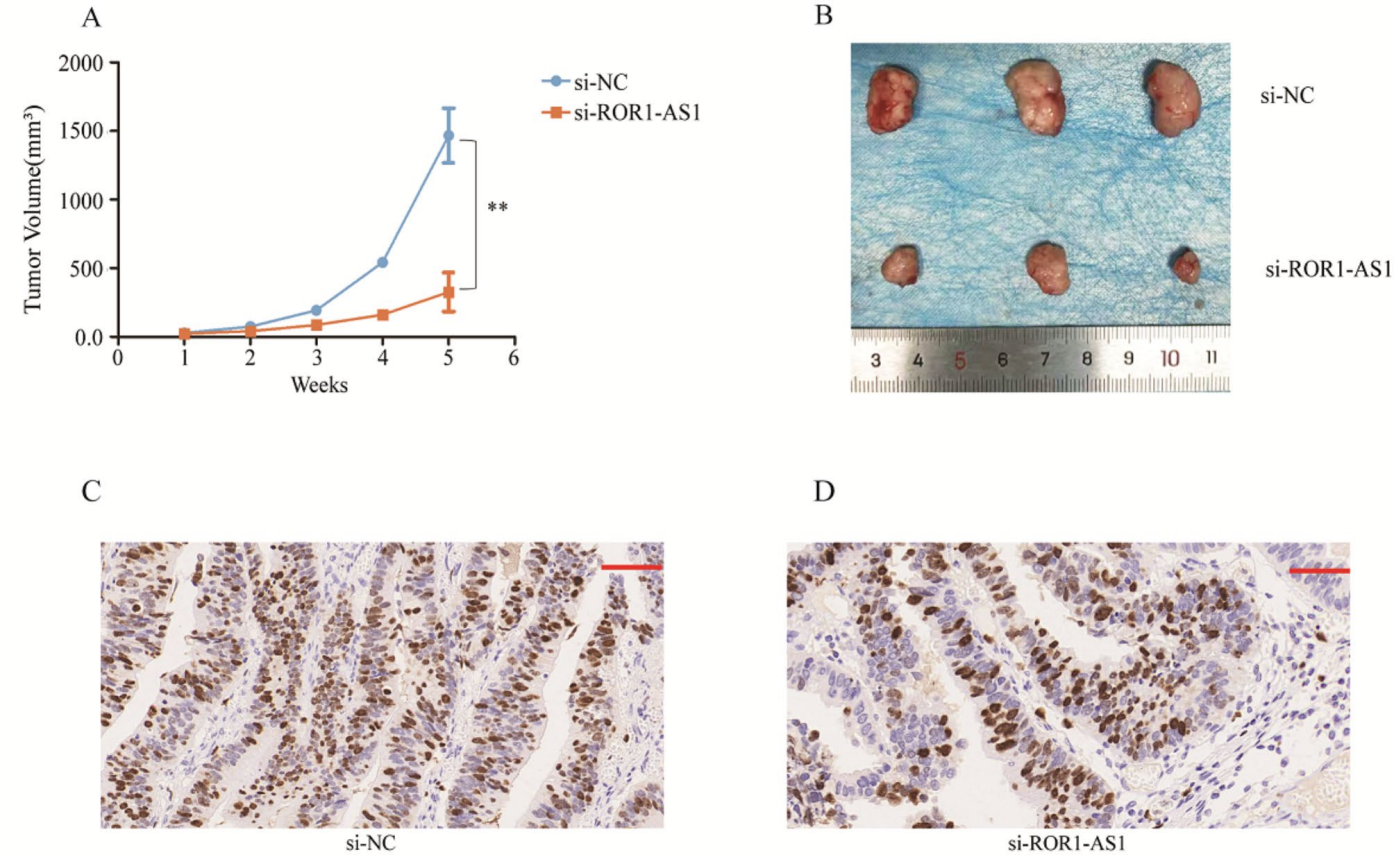
(**A-B**) The role of ROR1-AS1 in immunity and disease progression in animal experiments with cholangiocarcinoma cells. (**C-D**) Expression of Ki-67 protein was examined by immunohistochemistry in xenograft tumor tissues. Scale bars, 50 μm

To determine the effect of ROR1-AS1 on CCA cells, we divided the mice into two groups: an experimental group, which was administered ROR1-AS1 KD cells, and a control group, which received ROR1-AS1 cells. The results showed that tumor volume was reduced in the experimental group compared to that in the control group (Fig. 6A, B). Furthermore, our animal experiments fully demonstrated that ROR1-AS1 can promote the growth and proliferation of CCA cells. Moreover, expression of Ki-67 protein in the si-ROR1-AS1 group was significantly lower than that of the si-NC group(Figure 6 C, D).

### ROR1-AS1 mechanism in CCA

**Fig. 7:**
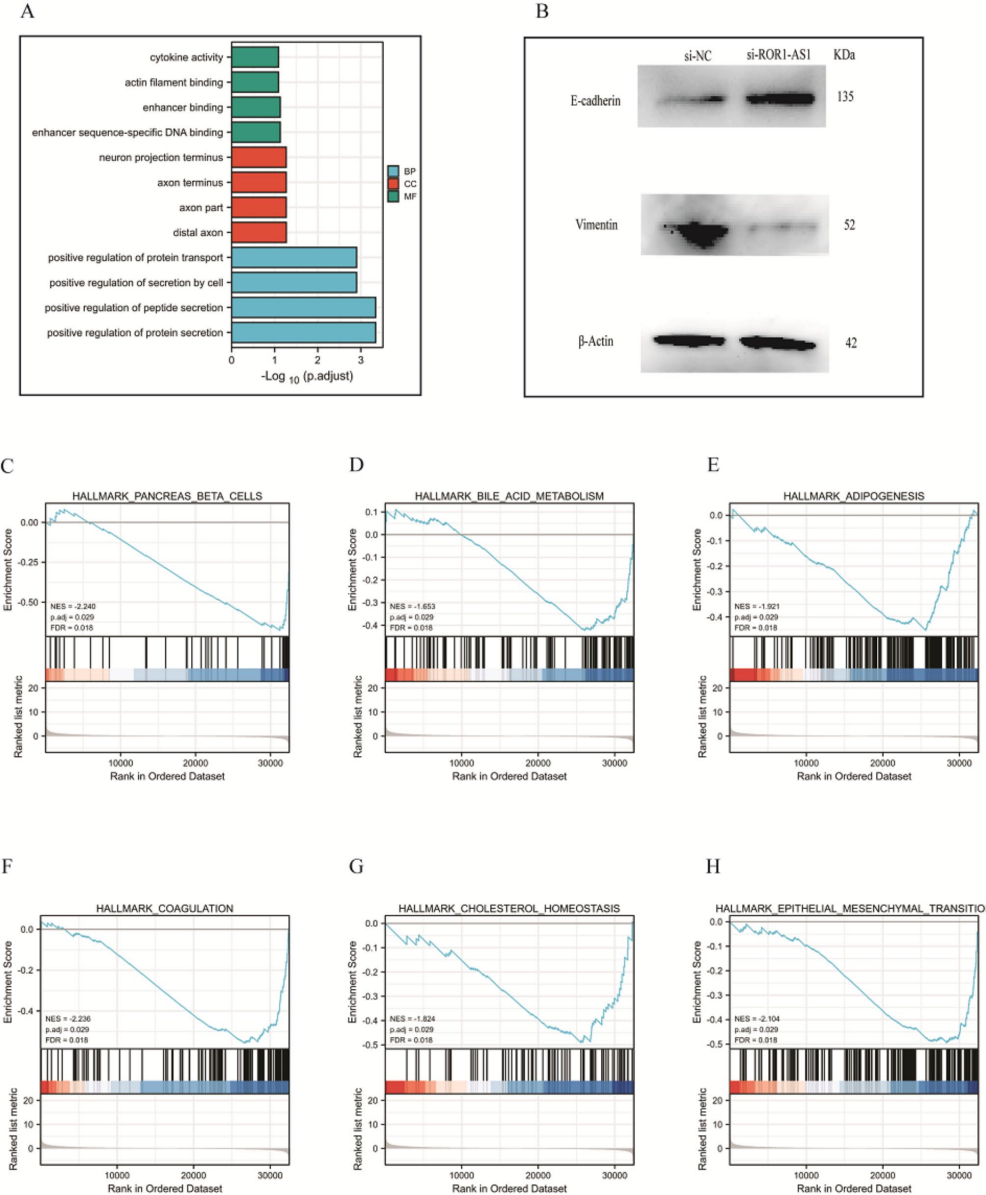
Functional enrichment of ROR1-AS1 in CCA. (**A**) GO enrichment analysis of differentially expressed genes in high- and low-ROR1-AS1 expression samples. (**B**) The protein levels of E-cadherin, Vimentin were detected by western blotting about ROR1-AS1. (**C**) GSEA of genes in the PBC signaling pathway. (**D**) GSEA of genes in the _BAM signaling pathway. (**E**) GSEA of genes in the ADIPOGENESIS pathway. (**F**) GSEA of genes in the COAGULATION pathway. (**G**) GSEA of genes in the CHOLESTEROL_HOMEOSTASIS. (**H**) GSEA of genes in the MET. CCA( cholangiocarcinoma), GO( gene ontology), GSEA( gene set enrichment analysis), MET(mesenchymal-to-epithelial transition)

To investigate the latent function of ROR1-AS1 in tumor development, we predicted the mechanism of co-expression in patients with CCA using GO analysis. The top GO terms were the great adjust of protein transport, good adjust of secretion by cell, positive regulation of peptide secretion, and active regulation of protein secretion. Other enrichment pathways were not significant, as shown in Fig. 7A. We also performed GSEA to identify the significant pathways relevant to ROR1-AS1 in CCA. In Fig. 7 C-H, the top six signaling pathways are listed in the hallmark database. The hallmark epithelial-to-mesenchymal transition (EMT) pathways in CCA were the most abundant hallmark signaling pathways. These analyses showed that ROR1-AS1 promotes the growth of CCA cells through certain pathways.

To further explore ROR1-AS1 effects on EMT in cholangiocarcinoma, we conducted the Western blot experiment. As we can see in Fig. 7B, the results show that compared to the NC group, ROR1-AS1 knockdown increased the relative expression levels of E-cadherin and reduced the expression level of Vimentin which are EMT marker proteins. It further confirms ROR1-AS1 the effect of EMT-related gene expression, and then explores the mechanism of ROR1-AS1 on cholangiocarcinoma.

**Fig. 8:**
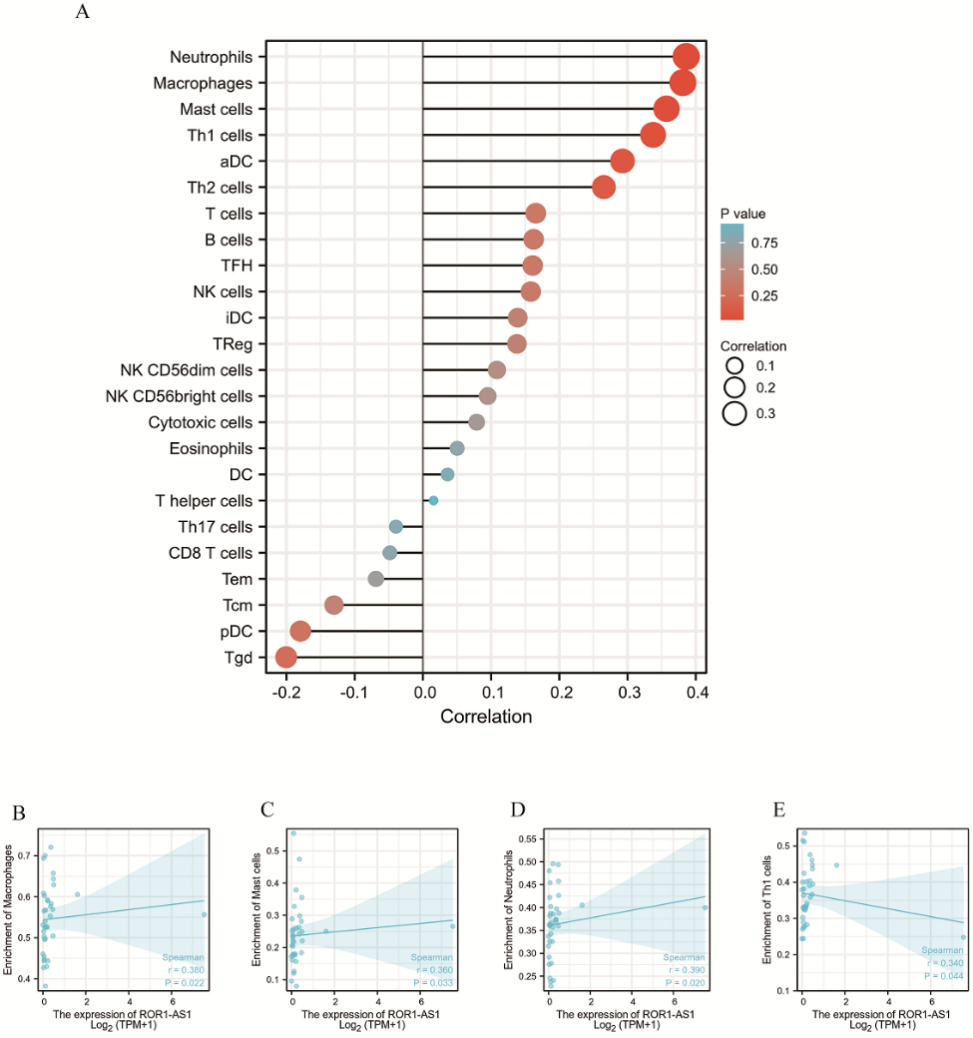
(**A**) The ratio of 23 subpopulations of immune cells, with macrophages, and aDC and Th1 cells being the main immune cells affected by ROR1-AS1 expression. Among them, resting macrophages cells (P = 0.043), aDC cells (P = 0.032) and Th1 cells (P = 0.047) are apparently increased in the high expression group compared with the low expression group. In contrast, activated NK cells (P = 0.46), T cells (P = 0.214) and other immune cells are decreased in the low expression group compared to high expression group. Therefore, other immune cells do not have an apparent relationship with ROR1-AS1 expression in CCA. (B-E) In contrast to those immune cells, resting macrophages (P = 0.022), mast cells (P = 0.032), neutrophils (P = 0.020), aDC cells (P = 0.031), Th1 cells (P = 0.044), and the four immune cells are closely correlated with ROR1-AS1 expression in CCA

We used CIBERSORT to predict the association between ROR1-AS1 expression and immune cell infiltration. For diverse immune infiltration levels, we selected the ROR1-AS1 expression levels that were significantly correlated with tumor purity. Our conclusions suggested a possible link between ROR1-AS1 overexpression and the immune and invasive capacities of CCA, as shown in Fig. 8A. Furthermore, we found that ROR1-AS1 may affect CCA progression by affecting cellular immune infiltration pathways and cell types, such as macrophage quiescence, mast cells, neutrophils, aDC cells, and Th1 cells (Fig. 8B, C, D, E). In conclusion, we can conclude that ROR1-AS1 may affect immune cell infiltration and growth of CCA cells.

## Discussion

We obtained data for ROR1-AS1 expression in CCA cases and carried out the first-ever analysis of the coding capacity of ROR1-AS1 in a large group of human CCA patients. Further genetic exploration showed that ROR1-AS1 expression was a biomarker of CCA. At the same time, we also found that ROR1-AS1 is related to the perineural invasion (PNI) and its expression. PNI is an independent risk factor for CCA and has been considered a worse prognostic factor in CCA [14]. In addition, a negative PNI has been considered a reference for postoperative chemotherapy in some studies [15].

To further verify the role of ROR1-AS1 in CCA, we performed in vitro experiments using CCA cell lines. Previously, the role of ROR1-AS1 in non-small-cell lung cancer, bladder cancer, and colon cancer cells has been previously demonstrated [16–18]. Some studies have also shown that lncROR can inhibit P3 activity by activating PI53K/AKT, promoting the proliferation of arsenite-transformed keratinocytes, and providing a new carcinogenic mechanism for arsenite-induced skin cancer [19]. We have also confirmed the function of ROR1-AS1 in promoting the progression of CCA cells through a series of cell and animal experiments, providing a guiding direction for subsequent research on CCA.

In addition, our findings indicate that, in CCA, different immune-related indicators and immune infiltration levels may be closely related to ROR1-AS1 expression. Therefore, ROR1-AS1 may have a latent effect on tumor immunity. Previous studies have shown that ROR1-CAR T cells inhibit tumor development in lung and breast cancers [20]. Therefore, our research offers new evidence for understanding the latent role of ROR1-AS1 in tumor immunology and its function as a tumor biomarker. Many studies have been conducted on macrophages, showing that these cells perform a series of regenerative roles in inflammation and healing to deal with acute injuries in tissues [21]. In terms of crosstalk with other tumor-infiltrating cells, mast cells related to the tumor have the capacity to change the tumor environment [21]. Several studies have reported ROR1-AS1 overexpression in mantle cell lymphoma) [22]. Therefore, resting macrophages, mast cells, neutrophils, aDC cells, and Th1 cells may have a positive influence on ROR1-AS1 expression in CCA.

The receptor tyrosine kinase ROR1 has been shown to play a role in therapeutical efficiency in treatments for CLL and multiple solid tumors and immunotherapy targeting ROR1 has showed significant improvements in preclinical and clinical studies [23]. Presently, there are several therapeutic methods targeting ROR-1, including specific monoclonal antibodies, modified T cells, miRNAs, and tyrosine kinase inhibitors [24–26].

## Conclusion

In summary, our in vitro and in vivo experiments confirmed that ROR1-AS1 promotes proliferation, invasion, and migration of CCA cells. Furthermore, our enrichment analysis revealed that ROR1-AS1 plays a significant role in the regulation of EMT and other pathways. Therefore, ROR1-AS1 can be a potential prognostic indicator in patients with CCA.

### Electronic supplementary material

Below is the link to the electronic supplementary material.

**Supplemental Data S1**: The list of animal studies

**Supplemental Data S2**. The qPCR primer sequence for ROR1-AS1.

## Data Availability

The datasetsanalysed during the current study are available in TCGA ((https://portal.gdc.cancer.gov/repository). All data and outcomes generated during this study are included in this published article and its supplementary information files. The data will be freely available to other researchers upon request for further interpretation and analysis. Then we present the qPCR primer sequence for ROR1-AS1 in Supplemental Data S2.
