# Supplementary material for: ROR1-AS1 might promote in vivo and in vitro proliferation and invasion of cholangiocarcinoma cells
Source: BMC Cancer. 2023 Sep 28;23:912. doi: 10.1186/s12885-023-11412-1 (PMC10536779; doi:10.1186/s12885-023-11412-1)
Supplement: Supplementary file 1 — Supplemental Data S1: The list of animal studies [file 12885_2023_11412_MOESM1_ESM.docx]

**Supplemental Data S1: The list of animal studies**

Study design and sample size

All mice were raised in a pathogen-free animal facility and randomly assigned to a control group or an experimental group (3 mice in each group). Where we performed the grouping scenario as follows: control we used cell lines that were not knocked out of ROR1-AS1, and experimental groups used cell lines that were knocked out of ROR1-AS1.

Inclusion and exclusion criteria and randomisation

Nude Mice (4 weeks old) were provided by Shandong Teke Biotechnology Company (Shandong, China). All mice were raised in a pathogen-free animal facility and randomly assigned to a control group or an experimental group.

Blinding

Neither the subjects nor the researchers were aware of the grouping, and the researchers arranged and controlled all the trials.

Outcome messures

1.The tumor growth rate in the experimental group was faster than that in the control group.

2.The tumor growth rate in the control group was faster than that in the experimental group.

3.The tumors grew equally fast in both groups.

4.The tumors did not grow in both groups.

Statiscial methods

All experiments were repeated at least three times, and representative data are shown in Fig.3, Fig.4 and Fig.5 right. However, the ultimate data are presented in Fig.3, Fig.4 and Fig.5 right through histogram used by GraphPad Prism 6.0 as the means±SD from three biological replicates.

Experimental animals

We selected 4-week-old male nude mice with an average body weight of 22g. Each mouse was healthy and there were no significant differences between the groups except for the control conditions. Each of our mice is the same without the slightest favoritism.

Experimental procedures

We then subcutaneously injected HuccT-1 cells about CHOL (3 × 10^6^) resuspended in 0.2 ml DMEM to the right side of each nude mouse. After completion of injection, we measured tumor changes every 3 days and calculated tumor volume as V = 0.5 × L × W ^2^(V=volume, L=length, W=width). After 35days, we killed the mice with anesthesia and euthanasia, and the tumor was removed. Then we compared the size of the tumor to determine the effect of the experiment.

Result.

To determine the promoting effect of ROR1-AS1 on cholangiocarcinoma cells, we divided mice into two groups, an experimental group in which ROR1-AS1 was knocked out and a control group in which ROR1-AS1 was not knocked out. The results showed that the tumor volume was reduced in the HuCCT-1 group compared with the control group (Figure 6A,B). And we also show that our animal experiments fully demonstrated that ROR1-AS1 can promote the growth and proliferation of cholangiocarcinoma cells.
