# Supplementary material for: ROR1-AS1 might promote in vivo and in vitro proliferation and invasion of cholangiocarcinoma cells
Source: BMC Cancer. 2023 Sep 28;23:912. doi: 10.1186/s12885-023-11412-1 (PMC10536779; doi:10.1186/s12885-023-11412-1)
Supplement: Supplementary file 2 — Supplemental Data S2. The qPCR primer sequence for ROR1-AS1. [file 12885_2023_11412_MOESM2_ESM.docx]

**Supplemental Data S2.** **The qPCR primer sequence for ROR1-AS1.**

| Gene ID | Species | Primer Sequence |
| --- | --- | --- |
| ROR1-AS1 | Human | Forward primer: 5’TCTTAGCAGGCATTTTGGAGG-3’ |
|  |  | Reverse primer: 5’-GCCAGGAAGTTTCAGCATTCTC-3’ |
